# Effect of unsteady cavitation on hydrodynamic performance of NACA 4412 Hydrofoil with novel triangular slot

**DOI:** 10.1016/j.heliyon.2025.e42266

**Published:** 2025-01-27

**Authors:** Sayan Biswas, R. Harish

**Affiliations:** School of Mechanical Engineering, Vellore Institute of Technology, Chennai, Tamil Nadu, 600127, India

**Keywords:** Passive flow control, Unsteady cavitation, SST *k-ω* turbulence model, Schnerr& Sauer model, NACA4412 hydrofoil

## Abstract

This study demonstrates the hydrodynamic performance of a modified NACA 4412 hydrofoil and compares it with the base NACA 4412 hydrofoil in the presence of cavitation. A triangular slot has been introduced at the mid-section of the suction side of the hydrofoil to modify the flow characteristics and assess its effects on the performance at different operational cavitation numbers spanning from 0.8 to 2.5, and at different angles of attack ranging from 4° to 16°. The performance metrics considered include the coefficient of lift, coefficient of drag, and the lift-to-drag ratio. The Reynolds number considered in this study is approximately 1.5 million. The SST *k-ω* turbulence model, homogeneous mixture multiphase model, and Schnerr& Sauer cavitation model have been employed in this study. At lower cavitation numbers, the modified hydrofoil is better able to control cavitation phenomena compared to the base hydrofoil. However, at higher cavitation numbers, the base hydrofoil exhibits slightly better performance than the modified hydrofoil in terms of lift-to-drag ratio, despite having a lower lift coefficient. Furthermore, the modified hydrofoil demonstrates improved performance at 4°, 8°, and 16° angles of attack, while the base hydrofoil performs better at a 12° angle of attack. It was also observed that stalling occurs at a 16° angle of attack for the base hydrofoil, whereas the modified hydrofoil successfully avoids stalling.

## 1 Introduction

Hydrofoils are lifting surface that are designed to reduce drag and increase the lift force on a vessel by generating a pressure difference between the upper and lower surfaces of the foil. Hydrofoils and bluff bodies of different shapes have been widely used in the marine industry to reduce drag, increase stability and efficiency of the vessels [1,2]. When a vessel is lifted onto hydrofoils, it experiences less water resistance compared to conventional hull designs. However, despite numerous advantages, the performance of hydrofoils is often limited by the onset of various physical phenomena such as cavitation and the presence of flow circulation, which can lead to increased drag and a reduction in lift [1]. Cavitation occurs as fluid pressure decreases beneath the vapor pressure, leading to the creation of vapor bubbles. These bubbles can collapse violently, causing damage to the hydrofoil surface and can create turbulent flow pattern, and cause localized pressure fluctuations, turbulence and vortices, which in turn can increase the overall drag experienced by the hydrofoil [2]. However, it is worth noting that super-cavitation can be helpful to reduce the drag as it protects the object by surrounding with cavitation bubble, instead of bubble collapsing. Vorticity, on the other hand, is a measure of the rotation of the fluid around the hydrofoil, and can also contribute to increased drag [3]. Therefore, the study of multiphase cavitation flow over hydrofoil has been under the spotlight of the researchers for a very long time. In the past, different methodologies have been proposed by different researchers considering analytical as well as experimental methods to study the hydrofoil performance in presence of cavitation [4]. However, with the development of numerical methods, it has been very popular method of study among the researchers to overcome the difficulties in analytical and experimental study of cavitation over hydrofoils.

Arndt et al. [5] performed experimental study using laser doppler anemometer to find out tip vortex generation characteristics on NACA66 series hydrofoil and reported a complex relation between cavitation behavior and considered parameters such as Reynolds number and gas content at the inception stage. They also mentioned that dependency of tip vortex trajectory on Reynolds number, cavitation number, angle of attack and dissolved gas content is not significant. Astolfi et al. [6] performed an experimental study to investigate the cavitation characteristics on a hydrofoil surface (Eppler E817) within a Reynolds number range of 0.4 × 10^6^ to 1.2 × 10^6^. The findings illustrated that both the magnitude and location of the lowest surface pressure coefficient, as ascertained from the velocity distribution in proximity to the leading edge, were subject to Reynolds number effects. Furthermore, it was observed that the pressure inside the cavitation bubble was closely aligned with or even closer than the vapor pressure of the liquid. Farhat et al. [7] investigated the bubble cavitation over NACA0009 hydrofoil experimentally and reported that the local vaporization process causes the formation of periodic bubble cavitation and the pressure beneath the bubble is always below the expected vapor pressure. Foeth et al. [8] conducted experimental study to investigate the phenomenon of sheet cavitation on a three-dimensional NACA0009 hydrofoil and focused on the development of attached sheet cavity and shedding of the cloud cavity in details.

Crimi [9] experimented on four semi-span hydrofoil models with four different sweep angles to investigate the impact of sweep angle on cavitation inception and hydrofoil deterioration. He mentioned a significant improvement in lift to drag ratio and drag at constant lift with increased sweep angle. Qin et al. [10] examined the dynamic behavior of cavitation and cavity induced vibration on NACA66 hydrofoil and reported that the maximum vibration amplitude remained relatively small during inception and sheet cavitation, increased significantly during cloud cavitation, and decreased during super cavitation. Wang et al. [11] also investigated the transient cavitation characteristics in four stages (i.e., no cavitation, inception, sheet and cloud cavitation) and cavity induced vibration over NACA66 hydrofoil. Liu et al. [12] established the correlation between cavitation and the oscillations in lift experienced by an S-shaped hydrofoil. In the cloud cavitation regime, the lift fluctuation is significantly higher than the other regimes. They also reported that once the cloud cavitation occurs, the cavity length increases faster. Simanto et al. [13] modified the leading edge of NACA0012 hydrofoil with three different types of sinusoidal protuberances in their experimental study of cavitation and reported the leading-edge protuberances are effective in order to reduce the cavity and as well as flow induced noise. Delgosha et al. [14] explored the impact of different irregularities on cavitation behavior on a 2-D foil and found out a reduction in cavity length with increase in surface roughness, leading to elevated oscillation frequency and disrupting the regular pattern of cavitation formation and collapse.

Zhang et al. [15] also investigated the effect of surface roughness of Clark-Y hydrofoil on its cloud cavitation characteristics using high speed video and particle image velocimetry and reported a significance influence of roughness on cavity pattern, velocity and vorticity distribution. Wang et al. [16] utilized high-speed video-graph and particle image velocimetry to examine ventilated cavitation flow structures. The results categorized the flow patterns into two distinct types: vortex shedding and relatively stable structures. Peng et al. [17] demonstrated tip vortex flow of a elliptical hydrofoil with NACA 662-415 section with and without cavitation and concluded that the air content has a huge impact on the cavitation incepting of tip vortex, mainly in case of low incoming velocity. Lin et al. [18] investigated fluid-structure interaction in cavitating flows around flexible and stiff NACA 0015 hydrofoils in a low-pressure tunnel. They revealed that flexible hydrofoils experience faster shifts in cavity shedding frequency and significantly higher structural vibrations in the cloud cavitation regime compared to stiff hydrofoils under similar cavitating conditions. However, it is very difficult to study cavitation flow experimentally due to several reasons such as requirement of specialized instrumentations to capture complex flow phenomena, ensuring reproducibility amidst boundary effects and sensitivity to various parameters.

To overcome the barriers of experimental study various numerical methods have been proposed and developed efficiently by various researchers throughout the years and being widely used in the cavitation study of a hydrofoil. Roohi et al. [19] performed a numerical investigation employing the Large Eddy Simulation (LES) technique and the Volume of Fluid (VOF) method to analyze cavitation effects on a two-dimensional Clark Y hydrofoil. The resulting predictions for lift and drag coefficient values exhibited a noteworthy agreement with experimental data. Ji et al. [20] also used LES model in their study on Clark Y hydrofoil. Movahedian et al. [21] studied the cavitation characteristics of a twisted 3-d hydrofoil using LES and VOF methods and reported that their simulation results such as cavitation patterns and the frequency of vortex shedding are in good agreement with experimental values. Similar models have been used by many more researchers in their study [[22], [23], [24]]. Wang and Maksoud [25] employed 2D remeshed vortex method with Brinkman penalization scheme to study the flow around 2D cylinder and foil at low Reynolds number and compared the obtained results with that of the available finite volume CFD solver. They reported that their proposed method is suitable for flow with far-field boundary conditions, but it provides only first-order accuracy with regard to time-step.

However, owing to the substantial computational expenditure and the intricacies involved in attaining grid independence through Large Eddy Simulation (LES), turbulence models based on the Reynolds-averaged Navier-Stokes (RANS) approach are extensively employed for the analysis of different cavitation phenomena. Peters et al. [26] employed an implicit RANS solver to simulate the cavitating flow around a ship propeller, utilizing the Volume of Fluid (VOF) method to model the interface between the two phases.

Ziru et al. [27] aimed to assess cavitation erosion risk n NACA0015 hydrofoil using RANS turbulence (SST k−ω model) method and fund that the obtained cavitation dynamics are in good agreement with experimental observations. Li et al. [28] used modified k−ω model (RANS based) and Schnerr-Sauer's cavitation model to predict the unsteady flows around 2D and 3D hydrofoils and found that the RANS model was able to predict the important features such as cloud cavities and re-entrant jet. Kadivar et al. [29] applied Partially-averaged Navier-Stokes (PANS) turbulence model, coupling with a mass transfer model in their study on unsteady cloud cavitation over CAV2003 hydrofoil and validated their findings with experimental results. Jin et al. [30] used RNG k-ε turbulence model in their study on a 2D NACA0015 hydrofoil. Zhao et al. [31] conducted a comparative assessment of the SST k-ω model, a modified variant of the SST k-ω model, and the LES-Smagorinsky model in the context of cavitation flow over a NACA0012 hydrofoil. Their research found that the modified SST k-ω model and the LES-Smagorinsky model were significantly more precise, yielding results that aligned more closely with experimental findings. On the other hand, various cavitation models have been developed by various researchers with time and Schnerr-Sauer [32], Kunz [33], Singhal [34] and Zwart [35] model are widely used among the researchers. With the development of various numerical methods, it has become easier to study different hydrofoils with various modifications, aiming to evaluate hydrofoil performance and analyze cavitation flow attributes. As a result, lot of research investigations has been reported successfully with geometry modifications of base hydrofoils as passive cavitation control technique.

Qun et al. [36] performed a numerical and optimization study on hydrofoil with slot in the presence of cavitation and reported that the optimized slot hydrofoil is showing better cavitation and hydraulic performances. Kadivar et al. [1,2] employed cylindrical cavitating-bubble generators to investigate and manipulate cavitation effects over hydrofoil and indicated an improvement in mitigating cavitation induced instabilities and vibrations. In their experimental study [37] unsteady cavitation, they showed that unstable cavitation and the pressure pulsations in the wake region of the hydrofoil were reduced by introducing hemispherical vortex generators on NACA16-012 hydrofoil. Furthermore, they [38] introduced wedge-type vortex generator over CAV2003 hydrofoil as passive control technique and demonstrated the effectiveness of this method in impeding the formation of cloud cavitation. They [39] also examined unsteady cavitation surge around a semi-circular leading edge flat plate and evaluated a passive flow control using a miniature wedge-type vortex generator. Their findings demonstrated effectiveness of this passive control in stabilizing cavitation, suppressing cavity instabilities, and reducing high-pressure pulsations induced by unsteady cavitation surge in the wake region. Capurso et al. [40] introduced three slots near the leading-edge connecting pressure and suction side as a passive control system and performed a comparison study in terms of vapor volume fraction, lift and drag coefficient. They found out significant reduction in vapor fraction, although the lift coefficient decreases. Liu et al. [41] also introduced slot at different location in a 3D Clark Y hydrofoil and reported that the generated jet flow can block the re-entrant jet and help to suppress the unsteady cavitation.

Zhao et al. [42] carried out their study using SST k-ω model and Zwart cavitation model considering bionic fin-fin structure on NACA0015 foil. They found that bionic fin provides a more stable flow with lesser turbulent kinetic energy and better control on vortex shedding near the wall region compared to base NACA0015 foil. Li et al. [43] demonstrated an improvement in hydrodynamics of Clark Y hydrofoil in presence of wavy leading-edge protuberances. Custodio et al. [44] experimented with hydrofoils featuring bioinspired wavy leading edges on NACA 634-021 hydrofoil. They considered different amplitudes and wavelengths of the edges in their study and found out that the hydrofoils with large amplitude possess better performance than the others; however, the baseline hydrofoils have better or similar performance than the hydrofoils with smaller amplitudes. Shi et al. [45] introduced a micro-channel in a NACA 0012 hydrofoil and reported reduction in cavitation generation and development. They also mentioned that the position and size of the micro-channel also plays an important role in its effectiveness. Cheng et al. [46] introduced overhanging grooves (OHGs) with winglets as a novel technique to suppress tip-leakage vortex cavitation on NACA0009 hydrofoils. OHGs outperform conventional methods, effectively mitigating cavitation across various gap sizes, showcasing promise for hydraulic machinery. Additionally, the study found minimal impact on the hydrofoil's drag and lift coefficients due to the overhanging grooves.

Lin et al. [47] numerically investigated the impact of arc obstacles of different sizes placed on the upper side of a flat hydrofoil and reported that cavity shedding decreases in presence of arc obstacle and also stabilize the frequency of shedding. Zhang et al. [48] performed both experimental and numerical study on cavitation shedding control by placing an obstacle on a hydrofoil surface and revealed a shift from large scale to small scale cavitation shedding due to the obstacle, inhibiting shedding dynamics and favoring a more manageable small-scale mode. Xu et al. [49] introduced a cavitator on the lower side of the NACA0012 hydrofoil and simulated its impact on super-cavitation regime. They mentioned a change in lift characteristics in presence of the cavitator, but the change in drag is minimal. Qiu et al. [50] examined the influence of micro-vortex generators on cavitation erosion on a hydrofoil and mentioned the reduction in the intensity of cavity, leading to decreased pressure fluctuation and acoustic power. VGs concentrate impact energy around cavity closure, resulting in a significant reduction in maximum impact energy compared to a smooth hydrofoil. Wang et al. [51] employed SST *k*-*ω* turbulence model with Zwart-Gerber-Belamri cavitation model to find out the effect of rectangular obstacle on cavitation characteristics over NACA0015 hydrofoil and their results indicated an increase in lift to drag ratio and the evolution of cavitation was suppressed effectively. Kumar et al. [52] compared the hydrodynamic performance of NACA 4412 base hydrofoil with dimpled NACA4412 hydrofoil considering different angle of attack, and found out a mixed performance result. However, they reported increases in lift in case of dimple hydrofoil at 16° angle of attack. Zeng et al. [53] studied the effect of cavitation on a vibrating hydrofoil, finding that resonance amplitude decreases with cavitation, reaching a 74 % reduction at inception. They also observed that added mass is linearly related to maximum cavity length, and hydrodynamic damping decreases by 39 % at high velocities when cavitation fully covers the hydrofoil. Zeng et al. [54] explored blade fatigue in turbines converting ocean energy, addressing the challenge of predicting resonance caused by varying flow velocities. They improved prediction accuracy by introducing correction terms for velocity, attack angle, and tip clearance, significantly reducing errors in resonance predictions for bending and torsional modes. Zhang et al. [55] analyzed cavitating flow around a NACA0009 hydrofoil using experiments and simulations, evaluating a correction model for turbulent viscosity. The model accurately captured cavitation shedding behavior, matching large eddy simulations while offering computational efficiency similar to the standard k-ε model. Tang et al. [56] studied the unsteady flow field of a NACA66 hydrofoil under critical stall conditions, identifying low-frequency oscillations caused by separation vortex interactions that affect pressure pulsation and lift coefficient at specific Strouhal numbers.

The preceding literature highlights the extensive investigation carried out by various researchers on the NACA hydrofoil series. Particularly, the NACA44 hydrofoil series is recognized for its advantageous capacity to control cavitation due to its substantial leading edge. Nonetheless, its sharp trailing edge contributes to vortex-induced vibration, thereby diminishing its hydrodynamic efficiency and structural resilience. While limited studies have addressed the NACA 4412 hydrofoil, both in the presence and absence of cavitation, some endeavors have explored passive control techniques to enhance its performance. However, a comprehensive exploration into the hydrodynamic performance of the NACA 4412 hydrofoil, featuring a triangular slot at its midsection and subject to unsteady cavitation, remains notably absent. Consequently, the primary purpose of this study is to investigate exhaustively how the addition of a triangular slot affects the hydrodynamic behavior of the NACA 4412 hydrofoil. This investigation will include an analysis of critical parameters, such as the lift coefficient, drag coefficient, and lift-to-drag ratio, for a variety of attack angles and cavitation scenarios. A comparative analysis of the performance characteristics of the unmodified base hydrofoil has been conducted.

## 2 Mathematical background

In the present study, the flow is incompressible, and a homogeneous mixture model has been employed to simulate multiphase flow. Homogeneous mixture model considers the velocities and pressures of both phases to be coupled and influenced by each other within the mixture. In this model, the governing equations for multiphase flow include mass and momentum conservation equations which are used to accurately describe the flow behavior and interactions between the different phases in the system. The turbulent flow characteristics are modeled using SST k-ω turbulence model. The governing equations for mass, momentum, and the homogeneous mixture model are discussed below.

(1) $$\frac{\partial \left(\rho_{m}\right)}{\partial t}+\nabla .\left(\rho_{m}{\vec{v}}_{m}\right)=0$$

(2) $$\frac{\partial \left(\rho_{m}\vec{v_{m}}\right)}{\partial t}+\nabla .\left(\rho_{m}\vec{v_{m}}\vec{v_{m}}\right)=-\nabla p+\nabla .\left[\mu_{m}\left(\nabla {\vec{v}}_{m}+\nabla {\vec{v}}_{m}^{T}\right)\right]+\rho_{m}\vec{g}+\vec{F}$$

Where n is the number of phases, ${\vec{v}}_{m}$ is velocity of the mixture, p is the pressure, $\mu_{m}$ is the viscosity of the mixture, $\vec{g}$ is the gravitational force, and $\vec{F}$ is body force. The mixture density $\rho_{m}$ and the vapor volume fraction α are related by eq. (3).

(3) $$\rho_{m}=\alpha \rho_{v}+\left(1-\alpha \right)\rho_{l}$$

Where α stands for vapor volume fraction, m, v and l stand for mixture, vapor and liquid phase respectively.

The shear-stress transport *k-ω* (SST *k-ω*) turbulence model has been employed in the present study, which combine the robust ad accurate formulation of the *k-ω* model for the near wall region with the free stream independence characteristic of the *k –*ϵ model for the far field. The SST k-ω turbulence model was employed in this study due to its ability to accurately capture boundary layer behavior and cavitation dynamics in flows with adverse pressure gradients. Previous studies [27,31,42,51] have demonstrated its reliability in evaluating the hydrodynamic performance of hydrofoils, making it well-suited for the objectives of this investigation.

Schnerr& Sauer [32] model has been adapted to model cavitation. The vapor volume fraction can be obtained from the transport equation, mentioned in eq. (4).

(4) $$\frac{\partial \left(\alpha \rho_{v}\right)}{\partial t}+\nabla .\left(\alpha \rho_{v}\vec{v}\right)=R_{e}-R_{c}$$

Where $R_{e}$ and $R_{c}$ represents the evaporation and condensation term respectively. $R_{e}$ and $R_{c}$ are computed using Schnerr & Sauer formulation as mentioned in equations (5), (6)).

When vapor pressure of the liquid $P_{v\geq }$ P,

(5) $$R_{e}=\frac{\rho_{v}\rho_{l}}{\rho }\alpha \left(1-\alpha \right)\frac{3}{R_{B}}\sqrt{\frac{2\;\left(P_{v}-P\right)}{3\;\rho_{l}}}$$

When $P_{v}\leq P$,

(6) $$R_{c}=\frac{\rho_{v}\rho_{l}}{\rho }\alpha \left(1-\alpha \right)\frac{3}{R_{B}}\sqrt{\frac{2\;\left(P-P_{v}\right)}{3\;\rho_{l}}}$$

Here $P_{v}$ is vapor pressure of the liquid, $R_{B}$ stands for bubble radius which can be related to α and bubble number density n_b_ as mentioned in eq. (7).

(7) RB=[(α1−α)(34πnb)]13

**Fig. 1:**
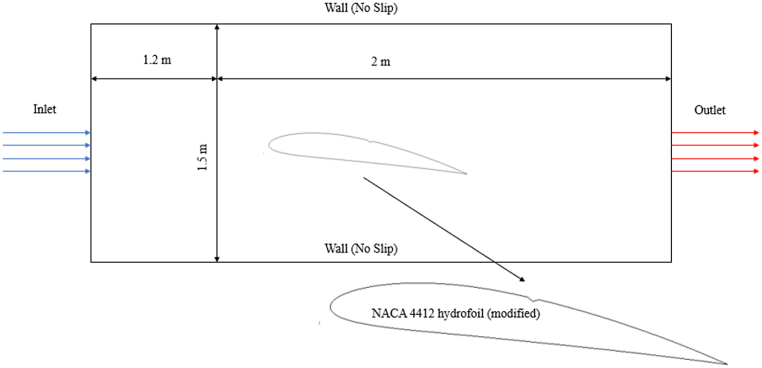
Schematic view of computational domain with boundary conditions.

In the present study, the inlet velocity has been taken as 18.3 m/s [57], and the outlet pressure has been calculated using eqn. 8.for different cavitation number. No slip boundary condition has been applied on the hydrofoil and other two walls of the computational domain (Fig. 1). The material properties of liquid and vapor phase have been considered from the available literature [57]: *ρ*_*l*_ = 999.2267 kg/m3, *μ*_*l*_ = 0.001175 Pa s, *ρ*_*v*_ = 0.01193 kg/m3, *μ*_*v*_ = 9.56 × 10^−06^ Pa s and the Reynolds number is around 1.556 million.

(8) $$\sigma =\frac{P-P_{v}}{0.5\times \rho \times v^{2}}$$

Here σ is the cavitation number, *P* is the local pressure of fluid flow and $P_{v}$ is the vapor pressure of the liquid, i.e., at which cavitation starts to occur.

### 2.1 Computational domain

Cambered NACA 4412 hydrofoils have been considered as the base hydrofoil in the present study. The considered modified hydrofoil has a triangular shape of cut/slot on the suction side. All the hydrofoils have a chord length of 0.1 m, camber ratio of 0.04, and thickness ratio of 0.12, a triangular cut/slot is introduced at a position 0.05 m away from the leading edge. The width of the slot cut is 3 mm and the depth is 0.75 mm. A rectangular computational domain having dimensions of 3.2 m × 1.5 m has been considered for the simulation of all the hydrofoils, where the inlet is 1.2 m away from the leading edge. The computational domain and the outline of the modified hydrofoil has been shown in Fig. 1.

### 2.2 Numerical setup

Fluid flow simulations were carried out utilizing Ansys Fluent [58], which employs the Finite Volume Method (FVM) for the numerical discretization of the governing equations of fluid flow. Pressure based transient simulations have been performed in the present study. The simulation utilized the SST *k-ω* turbulence model, well-known for its accuracy in predicting both boundary layer and free shear flow characteristics. Additionally, a Homogeneous Mixture Multiphase Model was applied, assuming a homogeneous mixing of phases to simplify the treatment of multiple phases' interactions. Liquid and vapor are considered as the primary and secondary phase respectively; and the properties are taken from the literature as mentioned in the mathematical background section. The Schnerr & Sauer cavitation model was also integrated to predict the formation and collapse of vapor bubbles within the flow, crucial for high-speed liquid flow applications. The Coupled Scheme facilitated pressure-velocity coupling, ensuring consistent interaction between pressure and velocity fields, while the PRESTO (Pseudo Transient Continuation) scheme was employed for pressure staggering, enhancing pressure computation accuracy in transient simulations. In the governing equations, the convection term is discretized using the second-order upwind scheme, while the diffusion terms are discretized using the central differencing scheme. The relative residual error criterion of 1 × 10^−6^ has been considered for assessing the convergence of mass, momentum, turbulent kinetic energy and turbulent dissipation rate.

### 2.3 Mesh convergence study

**Fig. 2:**
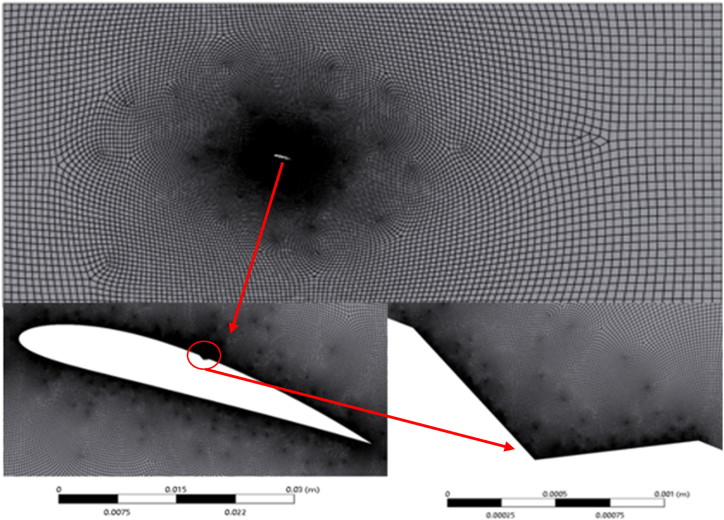
Meshing around NACA 4412 hydrofoil at 16⁰ angle of attack.

Mesh convergence study for NACA4412 base hydrofoil and the modified hydrofoil has been performed at an angle of attack of 16° and *σ* = 1, presented in Table 1. The generated mesh for hydrofoil is shown in Fig. 2.

**Table 1:** Mesh convergence study for NACA 4412 base hydrofoilat16⁰ angle of attack (σ = 1).

Grid	Mesh count	Mean lift coefficient over time
1	134824	1.651
2	176992	1.628
3	214209	1.627

From the presented data in Table 1, there are no substantial variations in lift coefficient between grid 2 and 3. Therefore mesh count of 1,76,992 has been considered for the rest of the study to save computational time and cost while maintaining good accuracy.

**Fig. 3:**
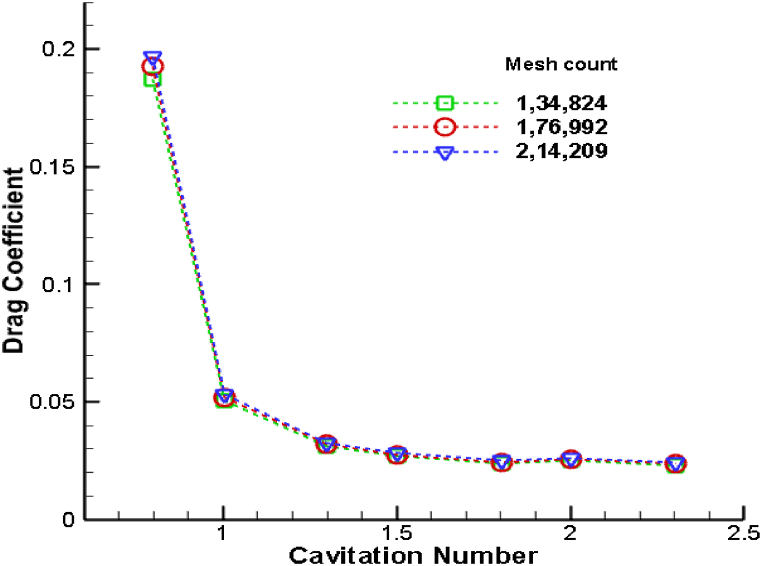
Variation of drag coefficient for different mesh size.

Furthermore, the mesh convergence study was continued with the help of time-averaged drag coefficient at 16**⁰** angle of attack at different cavitation number as shown in Fig. 3. The considered time-step size is 5 × 10^−3^ s. Fig. 3 illustrates the change in drag coefficient across three distinct mesh sizes. It becomes evident that the differences in drag coefficient values are marginal. The objective was to ascertain the impact of grid size on the accuracy of our simulation results, specifically focusing on the drag and lift coefficient as a critical parameter. In this study, a uniform time step size of 5 × 10^−3^ s was maintained across all grids. This is aimed to simplify the initial phase of analysis and establish a baseline for comparing the impact of grid size on the simulation results. Therefore, a mesh count of 1,76,992 has been adopted for all simulations conducted in this study.

### 2.4 Validation

**Fig. 4:**
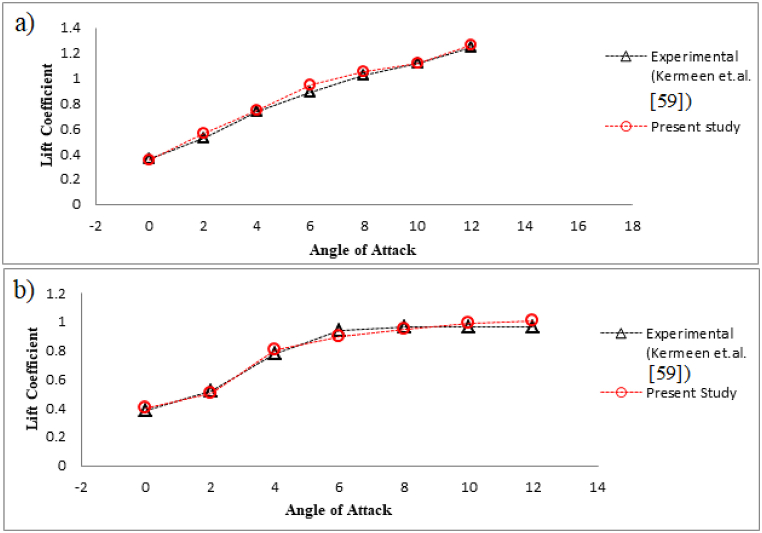
a) Validation study without cavitation and b) Validation study with cavitation.

The considered numerical model has been validated with the results obtained by Kermeen et al. [59], both with the cavitation at Re = 0.8 million and without cavitation at Re = 0.7 million. NACA 4412 hydrofoil with angle of attack ranging from 0° to 12° has been considered in both the cases. Kermeen et al. [59] preformed high speed water tunnel test on NACA4412 hydrofoil with two-dimensional section. Fig. 4(a) illustrates the lift coefficient comparison for NACA 4412 without cavitation, showing a consistent rise with higher angles of attack. This trend correlates with increased flow speed and curvature on the suction side and decreased values on the pressure side of the hydrofoil. Consequently, the pressure differential intensifies, resulting in a corresponding increase in the lift coefficient. The obtained lift coefficient in presence of cavitation (*σ* = 1) has been compared with the experimental values obtained by Karmeen et al. [59], as shown in Fig. 4(b).

From Fig. 4(b) it is can be observed that the lift coefficient increases with increase in angle of attack, although after a critical angle of attack, it remained almost same due to similar kind separation of flow on the suction side. Our numerical simulation predicted an upwards trend similar to the results obtained by experimental study of Kermeen et al. [59]. The experimental study used for validation involved a three-dimensional hydrofoil, while the CFD investigation focuses on a two-dimensional NACA 4412 hydrofoil with a triangular slot. Despite this difference, the lift coefficient trends, with and without cavitation at various angles of attack, align well with experimental data, confirming the two-dimensional model's ability to effectively capture core hydrodynamic behavior. Similar dimensional comparisons have been established in the literature to validate flow separation and cavitation behavior, particularly when the focus is on core dynamics rather than three-dimensional effects.

The quantitative comparison is mentioned in Table 2 and the highest percentage difference between our numerically obtained results and the experimental values obtained by kermeen et al. [59] are below 7 %, which is acceptable. Hence, it can be concluded that the examined numerical model exhibits favorable concurrence with experimental [59] outcomes, substantiating its suitability for subsequent investigations.

**Table 2:** Comparison of time averaged lift coefficient in presence of cavitation *σ* = 1.

Angle of Attack	0	2	4	6	8	10	12
Kermeen et al. [55]	0.38	0.52	0.78	0.94	0.96	0.96	0.96
Present Study	0.4	0.5	0.8	0.92	0.95	0.99	1.01
% Difference	5.26	3.84	2.56	2.13	1.04	3.13	5.21

## 3 Results and discussion

This study demonstrates hydrodynamic performance and cavitation phenomenon of NACA4412 hydrofoil with and without triangular cut. Lift and drag coefficient and lift to drag ratio has been considered with respect to different cavitation numbers and angle of attacks as the hydrodynamics performance evaluation metrics in the present study. The cavitation numbers are considered in range of 0.8–2.5 and four different angles of attacks are 4°, 8°, 12° and 16°.

### 3.1 Effect of cavitation numbers

**Fig. 5:**
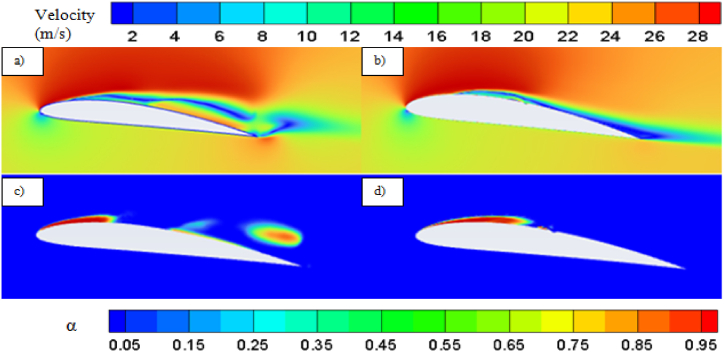
a) Velocity contour for base and b) modified hydrofoil. c) Vapor volume fraction for c) base and d) modified hydrofoil at *σ* = 0.8 (AoA = 8°).

Effect of cavitation regimes have been demonstrated for both base and modified hydrofoil at 8° angle of attack. The cavitation induced effects become more significant when vapor formation becomes favorable, particularly under conditions of lower cavitation number, as illustrated in Fig. 5. Hence stable vapor cavity forms on the suction side of the hydrofoil which helps to delay the flow separation as seem in Fig. 5 and favorable pressure distribution resulting in enhanced lift coefficient. As the vapor volume fraction over sucton side is high, the pressure drag increases, although the skin friction drag reduces. Due to the dominance of pressure drag, the overall drag coefficient is higher for both the hydrofoil at *σ* = 0.8.

**Fig. 6:**
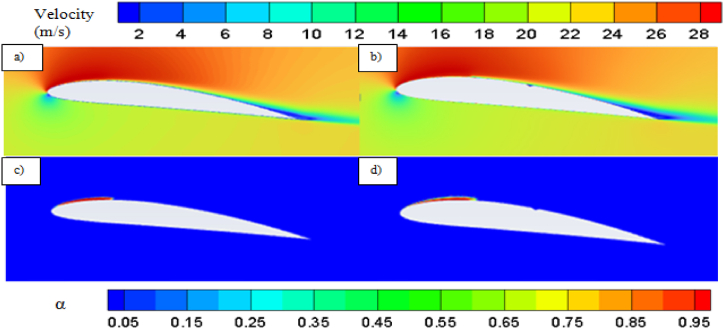
a) Velocity contour for base and b) modified hydrofoil, Vapor volume fraction for c) base and d) modified hydrofoil at *σ* = 1 (AoA = 8°).

**Fig. 7:**
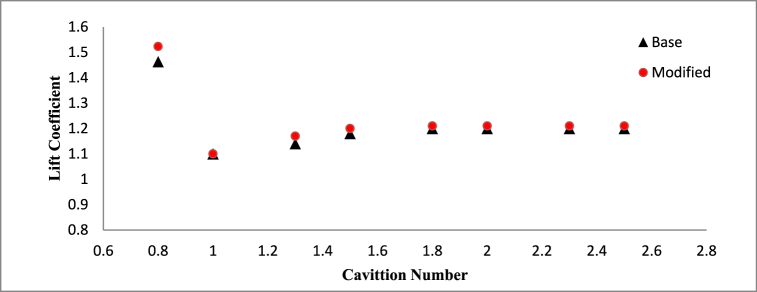
Mean lift coefficient plotted against the cavitation number for Angle of Attack = 8°.

**Fig. 8:**
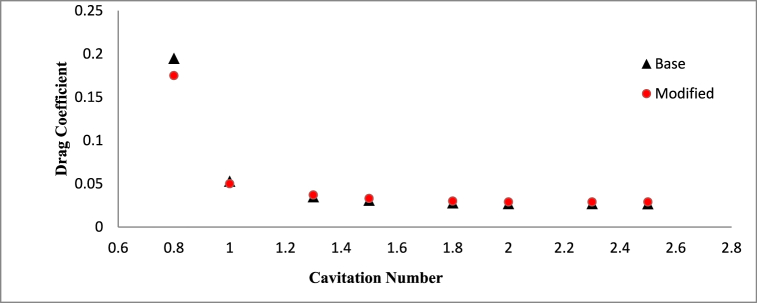
Time averaged drag coefficient vs Cavitation number (AoA = 8°).

With increase in cavitation number, at *σ* = 1, vapor cavity tends to collapse and formation of sheet cavity becomes salient in Fig. 6. The presence of sheet cavity leads to flow separation from the hydrofoil resulting in significant drop in lift coefficient. Also the pressure drag reduces significantly due to exiestence of lesser vapor over the hydrofoil. As the cavitation number increases, the sheet cavity length also decreases which leads to reduction in flow separation resulting in enhancement of lift coefficient gradually, as shown in Fig. 7. On the other hand, the reduced flow separation contributes to pressure recovery along the hydrofoil surface, further decreasing the pressure resistance, but the surface friction resistance increases gradually as more hydrofoil surface becomes open to the flow resulting in very small decrement in drag coefficient with higher cavitation number as shown in Fig. 8.

The sheet cavitation disappears eventually beyond a certain value of cavitation number, as result there is no significant interaction between cavitation and flow pattern around hydrofoil resulting in a constant lift and drag coefficient, which can be clearly observed in Fig. 7, Fig. 8.

**Fig. 9:**
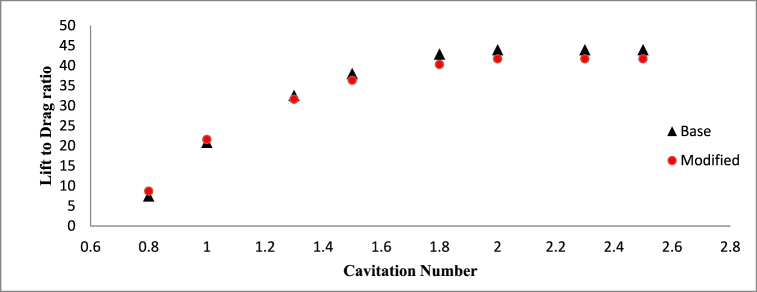
Lift to drag ratio variation for different Cavitation number for AoA = 8°.

Fig. 9 presents a comparative analysis of the lift-to-drag ratios for the base and modified NACA 4412 hydrofoils of *σ* = 1, AoA = 8°. The results highlight an upward trend in the lift-to-drag ratio with rising cavitation numbers, attributed to the elevation in lift coefficient and reduction in drag coefficient. Specifically, the modified hydrofoil exhibits a superior lift-to-drag ratio at lower cavitation numbers, while the base hydrofoil demonstrates improved performance in this ratio at higher cavitation numbers.

However, the difference between the lift to drag ratio for the considered hydrofoil is small. At lower cavitation number, the triangular cut at the mid-section of the hydrofoil has better control over cavitation characteristics and its interaction with surrounding flow which is clear from Fig. 9.

**Fig. 10:**
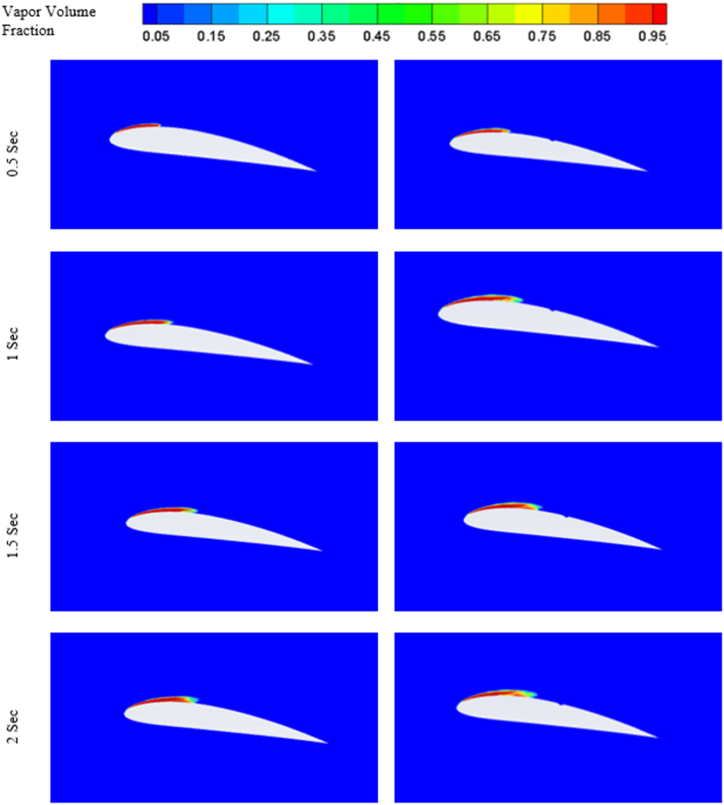
Cavitation at different times for 8° angle of attack (*σ* = 1).

Formation of cavitation over the hydrofoils at 8° angle of attack at four different time can be visualised from the figures mentioned in Fig. 10.

From Fig. 10, it can be observed that the cavitation effect is not much significant at lower angle of attack, as the similar kind of sheet cavity has been formed regardless of the simulation time. Although the cavity length slightly increases with increase in simulation time. Furthermore, the triangular slot doesn't have significant impact on the cavity formation at lower angle of attack.

### 3.2 Influence of angle of attack of the Hydrofoil

**Fig. 11:**
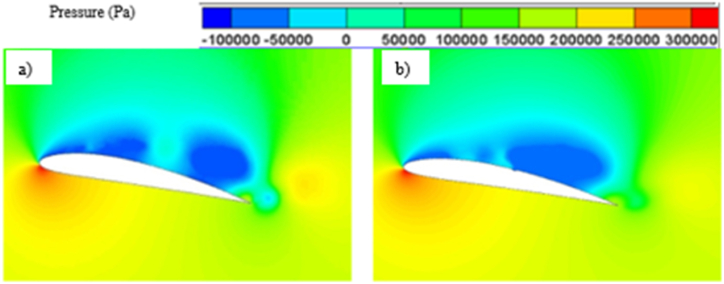
a) Pressure contour for Base and b) Modified hydrofoil at 12° angle of attack.

**Fig. 12:**
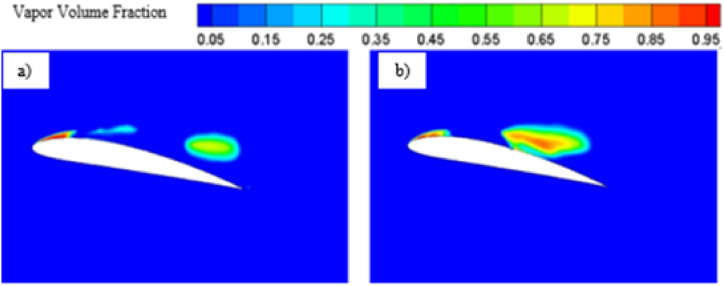
a) Vapor volume fraction for Base and b) Modified hydrofoil at 12° angle of attack.

**Fig. 13:**
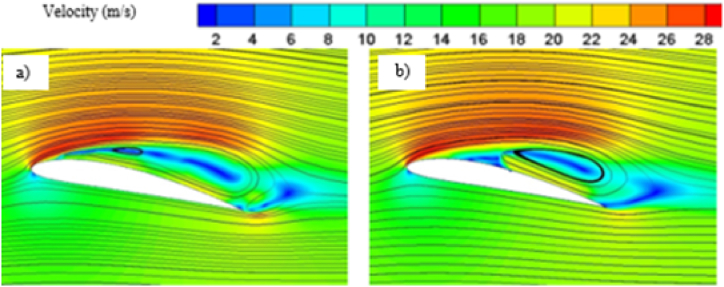
a) Velocity contour for Base and b) Modified hydrofoil at 12° angle of attack.

As the angle of attack increases, the pressure differential between the hydrofoil's suction and pressure side also intensifies. This phenomenon arises from the fluid flow's interaction with a steeper angle, resulting in heightened lift generation in accordance with Bernoulli's principle. Also, at higher angle of attacks, the circulation of flow increases due to the tendency of the separated flow to cover up the trailing edge of the hydrofoil, further contributing to the higher lift generation. On the other hand, both the skin friction drag and pressure drag increases as the flow separation starts to become more prominent with higher angle of attack. Skin friction mainly increases as flow separation comes closer to the leading edge causing an increase in portion of hydrofoil experiencing flow separation which leads to more resistance while moving along the surface. Pressure drags increases due to greater change in flow direction and formation of larger region of low-pressure wake or adverse pressure gradient which oppose the fluid flow. Beyond a certain angle of attack, lift coefficient starts to decrease and drag coefficient starts to increase rapidly, which is known as stalling. Stalling occurs due to excessive separation of flow and boundary layer. Formation of separated flow region also contributes in stalling. Stalling not only reduces the hydrodynamic performance of hydrofoil, it also reduces the stability and control of the whole system. The pressure contour, vapor volume fraction contour and velocity contour plot for higher angle of attacks i.e., 12° and 16° have been presented in Fig. 11, Fig. 12 and Fig. 13.

The pressure contour plots mentioned in Fig. 11 a) and 11 b), a more favorable pressure distribution can be observed on the suction side of the base hydrofoil. Again, larger vortex induces more turbulence resulting in more energy loss, which leads to higher drag coefficients for base hydrofoil than that of the modified one at 12° angle of attack. In case of 12°angle of attack, the leading-edge cavity is more or less similar for the base and modified hydrofoil, but the bubble cavity at the trailing edge of the base hydrofoil is smaller in size than that of the cavity at modified hydrofoil, leading to formation of a longer vortex extending from the front to the back edge over the base hydrofoil, as shown in Fig. 12 (a) and 12 (b). The cavity located proximate to the modified hydrofoil's trailing edge exhibits a partial attachment to the surface, resulting in a relatively larger size. This phenomenon contributes to the formation of reduced vortex dimensions spanning from the mid-section to the trailing edge, as visually depicted in Fig. 13(b). The longer vortex in base hydrofoil shown in Fig. 13 (a) leads in formation of favorable pressure gradients which improve the flow attachment and reduce the flow separation, resulting in increased lift. However, the flow separation is more and attachment is less in case of modified hydrofoil than that of the base hydrofoil which provides comparatively lesser lift generation.

**Fig. 14:**
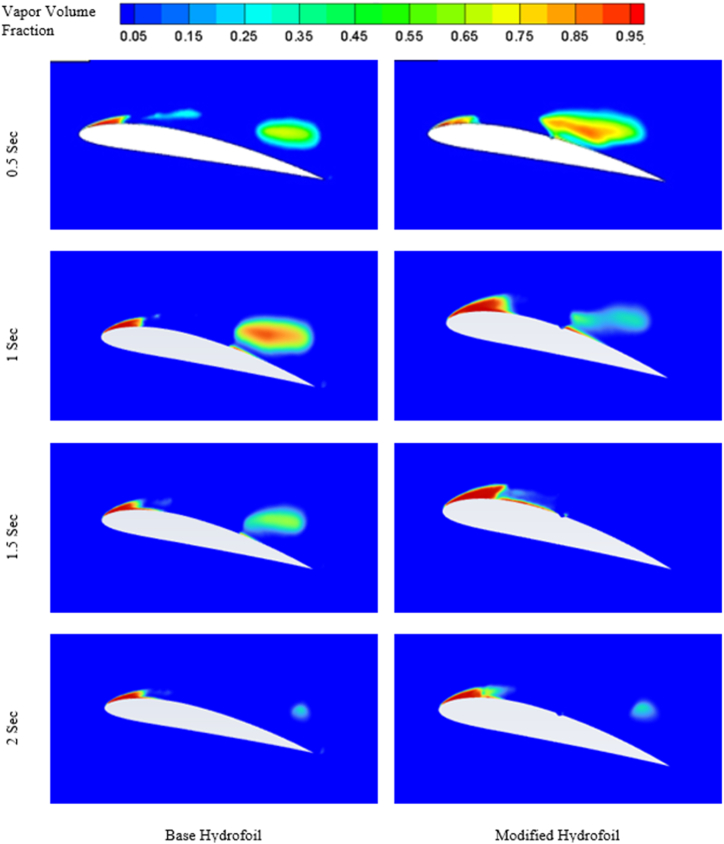
Cavitation at different times for 12° angle of attack.

The evolution of cavitation over both the hydrofoil at 12° angle of attack is presented in Fig. 14. At 0.5 s base hydrofoil has lower cavity volume over the suction side than that of the modified hydrofoil. The introduction of slot expedites the evolution cycle of cavitation. As the time increases, the volume of the cavity over the modified hydrofoil reduces than that of the base hydrofoil.

**Fig. 15:**
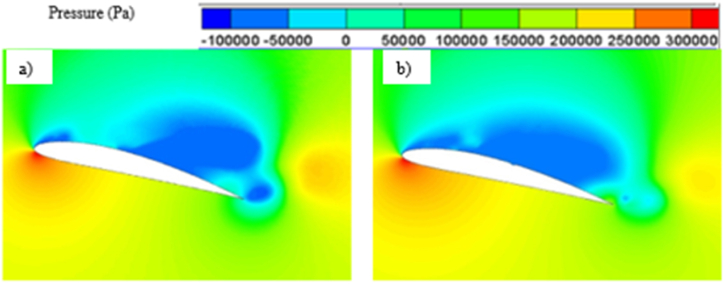
a) Pressure contour for Base and b) Modified hydrofoil at 16° angle of attack.

**Fig. 16:**
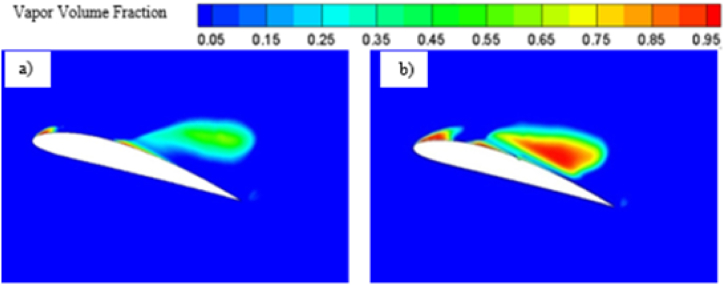
a) Vapor volume fraction for Base and b) Modified hydrofoil at 16° angle of attack.

**Fig. 17:**
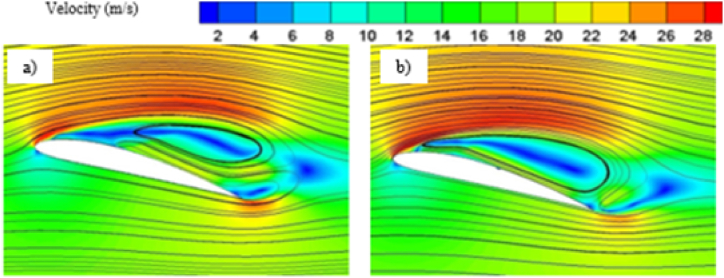
a) Velocity contour for Base and b) Modified hydrofoil at 16° angle of attack.

From the pressure contour, as presented in Fig. 15 a) and Fig. 15 b), it can be observed that the pressure distribution is more favorable in case of modified hydrofoil than that of the base hydrofoil at 16°angle of attack, resulting in higher lift generation in modified hydrofoil. It must be noted in this context that stalling takes place in case of base NACA 4412 hydrofoils at 16° angle of attack, although the modified hydrofoil can avoid stalling at 16°. The pressure contour plots in Fig. 11, Fig. 15 highlight how the slot influences the pressure distribution over the hydrofoil surface. At 12°, the slot induces a more uniform pressure gradient on the suction side, contributing to higher lift generation. As the angle of attack increases, the pressure differential between the suction and pressure sides intensifies. For the modified hydrofoil at 16°, the triangular slot enables a smoother pressure recovery on the trailing edge, as depicted in Fig. 15 (b). This smoother recovery reduces the low-pressure wake region, leading to a significant reduction in pressure drag. In contrast, the base hydrofoil exhibits a more abrupt pressure gradient and larger wake, resulting in higher drag and stalling tendencies. In case of base hydrofoil, there are two vortices formed over the hydrofoil, one medium sized vortex formed at a relatively higher position from the suction side and a small vortex with opposite rotation near the wake region. In case of 16° angle of attack, the vapor formation is different for each case as shown in Fig. 16 a) and Fig. 16 b), resulting in different flow characteristics i.e., pressure and velocity contour. Flow separation due to cavity formation is more in the base hydrofoil than that of the modified hydrofoil, which is shown with the help of velocity contour plot mentioned in Fig. 17 a) and Fig. 17 b).

**Fig. 18:**
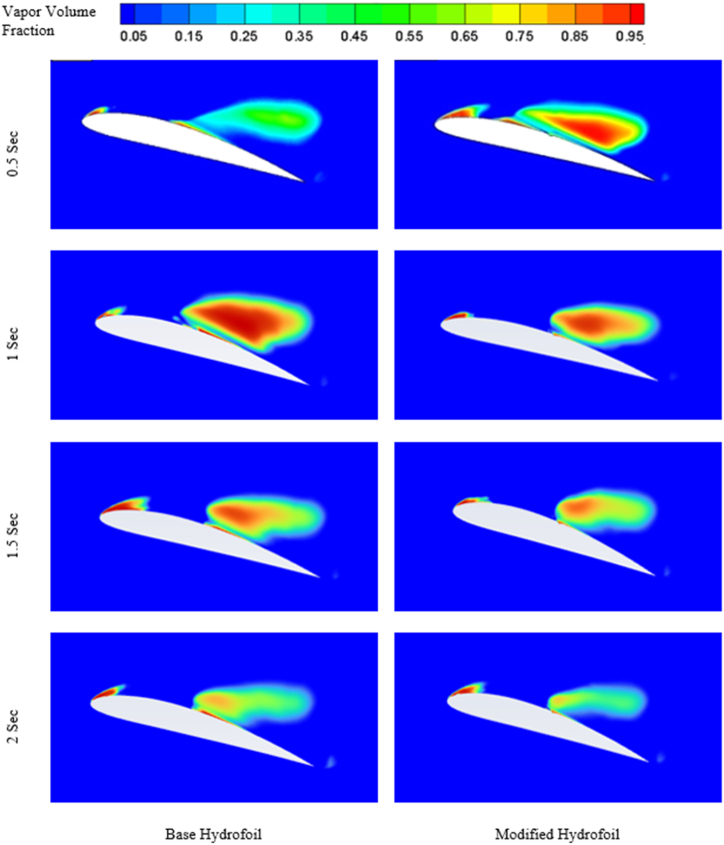
Cavitation at different times for 16° angle of attack.

The presence of two vortices indicates more complex flow pattern as it increases the mixing and energy loss. On the other hand, one full length vortex has been formed in case of modified hydrofoil which causes in relatively less complex flow pattern around the hydrofoil. The presence of single vortex induces lesser drag in modified hydrofoil due to lesser amount of mixing and energy losses than that of base hydrofoil with two vortices of opposite rotations as seen in Fig. 17 a) and 17 b). It should be noted that the flow separation in base hydrofoil is more than the modified hydrofoil and a greater number of vortices also affects the wake formation and leads to larger wake area, resulting in increased drag. The triangular slot on the modified hydrofoil plays a critical role in altering the boundary layer dynamics. At lower angles of attack, the slot helps to stabilize the boundary layer by reducing the adverse pressure gradient near the leading edge. This stabilization effect delays flow separation, as evident from the velocity contour plots in Fig. 17. At higher angles of attack, such as 12° and 16°, the boundary layer becomes increasingly prone to separation due to steep pressure gradients. The slot mitigates this effect by introducing controlled disturbances, promoting reattachment and delaying complete separation. This mechanism explains the modified hydrofoil's ability to avoid stalling at 16° when compared to the base hydrofoil. The introduction of the triangular slot significantly modifies the vortex formation dynamics around the hydrofoil. At 12°, the vortex structures for the base and modified hydrofoils differ notably, as shown in Fig. 13. The base hydrofoil exhibits a longer vortex extending from the leading to the trailing edge, which increases turbulence and energy losses. Conversely, the slot on the modified hydrofoil generates smaller, more localized vortices near the trailing edge. This localized vortex reduces the energy dissipation and improves the overall aerodynamic efficiency. At 16°, the velocity contour plots in Fig. 17 indicate the presence of two vortices of opposite rotation in the base hydrofoil, leading to complex flow patterns and increased drag. In contrast, the modified hydrofoil forms a single, elongated vortex that minimizes mixing losses and contributes to reduced drag. The evolution of cavitation over both the hydrofoil at 16° angle of attack is presented in Fig. 18. At 0.5 s, the cavitation volume is higher in case of modified hydrofoil than that of the base hydrofoil. However, with time the cavitation volume over modified hydrofoil has been reduced more than that of the base hydrofoil.

**Fig. 19:**
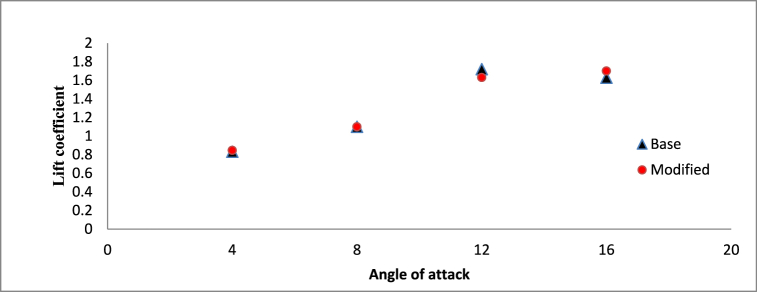
Time averaged lift coefficient vs Angle of attack (σ = 1).

**Fig. 20:**
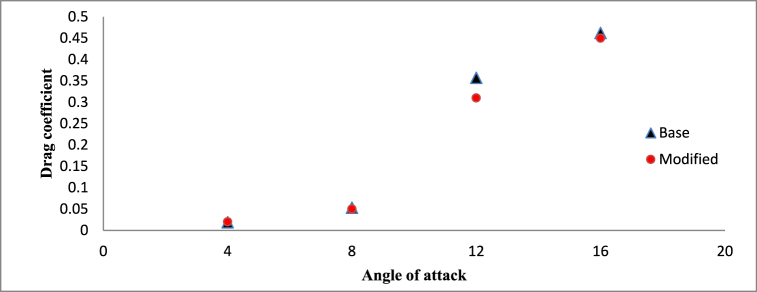
Time averaged drag coefficient vs Angle of attack (σ = 1).

From Fig. 19, Fig. 20, it can be observed that at lower angle of attacks of 4° and 8°, both the hydrofoils are having almost same lift and drag coefficient as the cavitation phenomenon is less prominent at lower angle of attack. But at higher angle of attack, tendency of cavitation increases significantly, resulting in complex flow characteristics around both the base and modified hydrofoil which causes significant variance in hydrodynamic performance.

**Fig. 21:**
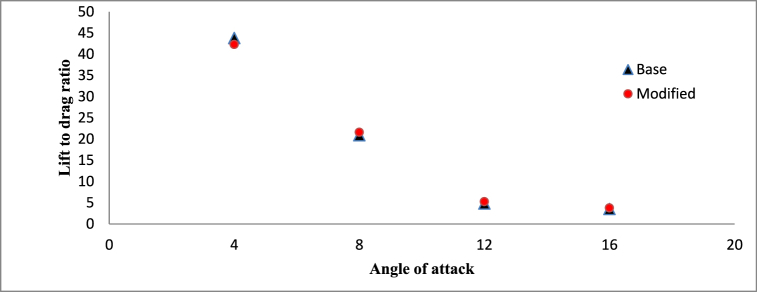
Lift to drag ratio vs Angle of attack (σ = 1).

The analysis of the lift-to-drag ratio between the base and modified hydrofoils is illustrated in Fig. 21. An observed trend indicates a decrease in the lift-to-drag ratio with an increase in the angle of attack. Specifically, at a lower angle of attack of 4°, the base hydrofoil demonstrates a slightly superior lift-to-drag ratio. Conversely, at the remaining three angles of attack, the modified hydrofoil showcases enhanced performance in the lift-to-drag ratio. It is noteworthy that, despite possessing a lower lift coefficient, the modified hydrofoil achieves a higher lift-to-drag ratio at 12°, owing to its reduced drag coefficient in comparison to the base hydrofoil. The triangular slot acts as a passive flow control mechanism, influencing multiple hydrodynamic characteristics. By stabilizing the boundary layer, moderating pressure distribution, and optimizing vortex dynamics, the slot enhances the hydrofoil's performance, particularly at higher angles of attack. These effects are most prominent at 12° and 16°, where the modified hydrofoil demonstrates superior lift-to-drag ratios due to its reduced drag coefficient.

## 4 Conclusions

A comparison study of hydrodynamics performance of base and modified NACA 4412 hydrofoils in presence of cavitation at different angle of attacks has been elucidated in this paper. RANS based turbulence mode and Schnerr-Sauer's cavitation model has been considered for the 2D transient simulations at various cavitation number and angle of attacks. Different complex flow behavior and their effects on the hydrodynamics performance of both the hydrofoil have been demonstrated in this study. Some notable findings among all the others can be concluded as.

• The modified hydrofoil demonstrates a notable enhancement, exhibiting a 4.1 % increase in lift alongside a 10 % reduction in drag coefficient when compared to the standard hydrofoil. These improvements are particularly pronounced at a lower cavitation number of σ = 0.8 and an angle of attack of 8°. Consequently, the incorporation of the slot has led to a significant enhancement in the lift-to-drag ratio, marking a substantial 15.6 % advancement in performance.
• With increases in cavitation number, the hydrodynamic performances of both the hydrofoil becomes almost same to each other, however, base NACA 4412 hydrofoil shows slightly better performance at higher cavitation number beyond σ = 1.5.
• At a cavitation number of 1, the hydrodynamic behavior of both the hydrofoil at a 4° angle of attack demonstrates close resemblance, owing to the absence of a cavity. Nevertheless, as the angle of attack increases to 8° and beyond, the emergence of cavitation becomes prominent, giving rise to intricate flow patterns that result in distinct hydrodynamic responses. Specifically, at an angle of attack of 8°, the adjusted hydrofoil exhibits a noteworthy enhancement in its lift-to-drag ratio, marked by a 3.35 % improvement.
• At 12° angle of attack and cavitation number of 1, the base hydrofoil shows better performance than modified one in terms of lift coefficient. But the modified hydrofoil produces almost 13.2 % lesser drag coefficient and 8.9 % more lift to drag ratio than the base hydrofoil.
• When subjected to a cavitation number of 1 and positioned at a 16° angle of attack, the adapted hydrofoil showcases a reduction of 2.6 % in drag as compared to the conventional hydrofoil. In addition, it generates a notable increment of 4.42 % in lift and an appreciable enhancement of 7.4 % in the lift-to-drag ratio relative to the base hydrofoil. Notably, the modified hydrofoil effectively prevents the onset of stalling at the 16° angle of attack, distinguishing itself from the base hydrofoil which experiences stalling under similar conditions.

This study investigated the unsteady cavitation dynamics and their impact on the hydrodynamic performance of a NACA 4412 hydrofoil equipped with a triangular slot. While the findings provide valuable insights, a comparative analysis with other passive flow control techniques, such as dimples or vortex generators, was not included. Future research exploring these alternative configurations under similar operating conditions would offer a clearer understanding of their relative effectiveness. Expanding the scope in this manner would enhance the applicability of the current results and deepen the understanding of passive flow control mechanisms in hydrodynamic systems. Although this study primarily focuses on 2D simulations, extending the analysis to 3D simulations could yield additional insights. In a 3D context, the triangular slot may improve spanwise flow control and enhance cavitation suppression across the entire hydrofoil surface. However, implementing the slot on a 3D hydrofoil presents challenges, such as increased computational resource requirements and the need to address potential impacts on secondary flows. These challenges highlight a critical area for future research, which could refine the understanding of the slot's effects in practical applications. The 2D simulation framework may not fully capture the complexities of real-world hydrofoil performance, and further investigation is needed to assess the applicability of the results to different hydrofoil designs and operating conditions. Variations in Reynolds number, cavitation number, and hydrofoil geometry could influence the effectiveness of the triangular slot, warranting additional study.

Future studies will also incorporate experimental validation through advanced flow visualization techniques, such as Particle Image Velocimetry and Laser-Induced Fluorescence, to capture detailed cavitation patterns and flow structures. These experiments will complement the numerical findings and enhance the predictive accuracy of the computational model. Additionally, a systematic parametric study of triangular slot variations, considering position, dimensions, and shape, will be undertaken to assess their impact on the hydrofoil's hydrodynamic performance under both cavitating and non-cavitating conditions. This analysis will identify key design parameters for optimizing cavitation control. A multidisciplinary approach combining fluid mechanics and materials science will also be used to assess cavitation effects, while the evaluation will be expanded to include noise levels, vibration characteristics, and material responses to cavitation-induced stresses. This comprehensive approach will contribute to hydrofoil design optimization.

**Table:** Nomenclature

*ρ*_*l*_	Density of liquid (Kg/m^3^)
*μ*_*l*_	Dynamic viscosity of liquid (Kg/(m.s))
*μ*_*v*_	Dynamic viscosity of vapor (Kg/(m.s))
g	Gravitational acceleration (m/s^2^)
σ	Cavitation number
$P_{v}$	Vapor pressure of liquid (Pa)
c	Chord length of hydrofoil (m)
A	Area of hydrofoil (m^2^)
C_l_	Lift coefficient
C_d_	Drag coefficient
C_p_	Pressure coefficient
k	Turbulent kinetic energy (m^2^/s)
ω	Turbulent dissipation rate (s^−1^)
Re	Reynolds number
V	Free-stream velocity (m/s)
t	Time (s)

## CRediT authorship contribution statement

**Sayan Biswas:** Writing – original draft, Visualization, Validation, Methodology, Investigation, Formal analysis, Data curation. **R. Harish:** Writing – review & editing, Supervision, Software, Project administration, Conceptualization.

## Data availability

Data available on request from the authors.

## Funding

There are no funding sources associated with this work.

## Declaration of competing interest

The authors declare that they have no known competing financial interests or personal relationships that could have appeared to influence the work reported in this paper.
